# Assessing the Impact of COVID-19 on Overdose Presentations Through the Emergency Department in a Large Tertiary Hospital

**DOI:** 10.1192/bjo.2022.480

**Published:** 2022-06-20

**Authors:** Raneem Saleh, Abdubadie Kutubi, Zainab Shobowale, Renad Saleh, Allys Guerandel, Susan Moore

**Affiliations:** St Vincent's University Hospital, Dublin, Ireland

## Abstract

**Aims:**

The outbreak of COVID-19, lockdown and self-isolation has created a lot of additional pressure on the society as a whole. We aim to audit the number of patients presenting to SVUH ED since March 27th 2020 (the date at which the government imposed a stay-at-home order) with an overdose.

**Methods:**

The cohort of cases analysed was identified using ED MAXIMS under the subheading of ‘overdose and poisoning’ presentations. Data were collected using MAXIMS and clinical portal and stored on the SVUH system and analysed using Microsoft Excel and SPSS.

**Results:**

There were a total of 713 cases in both years (from 27th of March – 31st December), with 353 (49.5%) admitted in 2019 and 360 (50.5%) admitted in 2020. Out of those admitted, 423 patients were females (196 and 227 in 2019 and 2020 respectively). There was a significant increase in the number of female presentations in 2020, with a p value of 0.041.

When stratifying patients based on age, the mean ages were 37.22 (SD 17.04) and 34.18 (SD17.32) in 2019 and 2020 respectively (p = 0.076). When dividing age groups in three categories (under 18, over 65 and 19–64), our data showed significant differences. There was a significant increase in numbers in the ≤18 yr and 19–64 age groups in 2020 compared with 2019. In the under 18 groups, there was an increase in numbers by 7.9% in 2020 (11.6% compared with 19.5%). When comparing numbers between Months per year, overall, there were no changes in presentations. Interestingly March 2020 had no presentations compared with March 2019, coinciding with the beginning of the pandemic in Ireland. May showed more than 50% decrease in presentations in 2020. Similar numbers were seen in the rest of the months of both years.

**Conclusion:**

Between 2019 and 2020, there was a 1.9% increase of ED presentations with overdoses, which did not show any significance in increase of numbers or in age demographics. There were three main findings from our analysis:

- *1.* A significant difference between the two years in a rise in female patients admitted with overdose (p = 0.041)
- *2.* An increase in presentations in the age groups of under 18 and 19–64. This may allude to increase strain in the younger population with loss of jobs, financial burdens etc.
- *3.* There were no presentations in March 2020, coinciding with the beginning of the pandemic in Ireland.

